# Is online learning during the COVID-19 pandemic associated with increased burnout in medical learners?: A medical school’s experience

**DOI:** 10.1371/journal.pone.0285402

**Published:** 2023-05-05

**Authors:** Sarah Hunt, Jenna Simpson, Lyndon Letwin, Bryan MacLeod

**Affiliations:** Northern Ontario School of Medicine (NOSM) University, Thunder Bay, ON, Canada; Alexandria University Faculty of Nursing, EGYPT

## Abstract

**Introduction:**

The COVID-19 pandemic necessitated a shift to virtual curriculum delivery at Canadian medical schools. At the NOSM University, some learners transitioned to entirely online learning, while others continued in-person, in-clinic learning. This study aimed to show that medical learners who transitioned to exclusively online learning exhibited higher levels of burnout compared to their peers who continued in-person, clinical learning. Analysis of factors that protect against burnout including resilience, mindfulness, and self-compassion exhibited by online and in-person learners at NOSM University during this curriculum shift were also explored.

**Methods:**

As part of a pilot wellness initiative, a cross-sectional online survey-based study of learner wellness was conducted at NOSM University during the 2020–2021 academic year. Seventy-four learners responded. The survey utilized the Maslach Burnout Inventory, the Brief Resilience Scale, Cognitive and Affective Mindfulness Scale–Revised, and the Self-Compassion Scale–Short Form. T-tests were utilized to compare these parameters in those who studied exclusively online and those who continued learning in-person in a clinical setting.

**Results:**

Medical learners who engaged in online learning exhibited significantly higher levels of burnout when compared with learners who continued in-person learning in a clinical setting, despite scoring equally on protective factors such as resilience, mindfulness, and self-compassion.

**Conclusion:**

The results discussed in this paper suggest that the increased time spent in a virtual learning environment during the COVID-19 pandemic might be associated with burnout among exclusively online learners, as compared to learners who were educated in clinical, in-person settings. Further inquiry should investigate causality and any protective factors that could mitigate negative effects of the virtual learning environment.

## Introduction

The novel coronavirus disease (COVID-19) pandemic caused innumerable changes to workplaces and educational institutions across the globe. Public health efforts to control the pandemic led to strict regulations on gathering sizes and social interactions [1]. These public restrictions and a move to a virtual learning environment introduced new stressors for medical students and residents, already engaged in a rigorous training program [2]. Medical students expressed distress regarding the COVID-19 situation, and indicated that the move from an in-person, interactive learning environment to online learning led to feelings of unpreparedness and a sense of deficiency in their skills [3–6].

Though the COVID-19 pandemic has persisted for years, there are very few studies that analyzed the impacts of online curriculum delivery on medical learners. A 2020 study of Swiss medical students and residents found higher levels of burnout among non-involved students who transitioned to online learning, while students who remained involved in healthcare delivery experienced less burnout than their peers [7]. The investigators postulated that these differences were due to difficulties adapting to the online learning environment, concern about assessment performance, and increased isolation, and that involvement in pandemic response was a protective factor [7].

A study of Polish medical students found similar results. Medical learners were transitioned to online learning, then surveyed using validated resilience, well-being, and burnout inventories [8]. Students that volunteered, worked, or planned to work on frontline pandemic response showed lower levels of burnout including exhaustion, lower cynicism, higher academic efficacy, compared to students who transitioned to online learning [8].

Efforts to better contextualize the thoughts and feelings of Canadian medical students throughout the pandemic via validated means have been limited, leaving room for further investigation. Other publications have explored the pandemic’s effects on global medical students, but comparisons between exclusively online learners and learners who continued learning in-person in clinic in Canada have not been made [5].

The data for this publication was initially collected and analyzed as part of a longitudinal study on the effectiveness of a voluntary mindful self-compassion training for medical learners at NOSM University. Prior to the mindful self-compassion course, learners participated in a pre-course survey measuring various metrics of wellness such as mindfulness, self-compassion, resilience, and burnout. The initial intention of these baseline measurements was to make comparisons following completion of the mindful self-compassion course. However, the data collection was initiated during and continued throughout the COVID-19 pandemic, which provided a unique opportunity to collect and analyze wellness data during this unprecedented period of change to the learning environment.

Based on the observations reported in the Swiss and Polish studies discussed above [7, 8], the hypothesis was that NOSM University medical learners who were exclusively online during the COVID-19 pandemic would exhibit higher levels of burnout than those who continued in-person, clinical learning during the COVID-19 pandemic. The first study objective was to demonstrate that there were higher levels of burnout among exclusively online medical learners, compared to learners who continued in-person. The second objective was to examine factors that protect against burnout, including self-compassion, mindfulness, and resilience, to detect any differences in protective traits between these two groups that may help further explain observed differences in burnout. The overall intention of this study is to provide further evidence of the mental health impacts of online learning on medical learners in the context of the COVID-19 pandemic, thereby contributing to a growing body of literature that furthers our understanding of the consequences of COVID-19 on medical education.

## Methods

A cross-sectional online survey-based study that measured learner well-being was conducted at NOSM University during the 2020–2021 academic year. Ethics approval was obtained by both Lakehead (#1468263) and Laurentian (#6020865) Universities prior to study commencement and was reaffirmed for the publication of this incidental finding. Learners were invited to complete the surveys via email invitations and word-of-mouth. Written consent was obtained from each of the participants via electronic consent forms. Learners who consented to the study completed the surveys on a voluntary basis.

### Questionnaires & survey measures

The online survey was built using Google Forms. The Maslach Burnout Inventory [9] was used to measure symptoms of burnout including emotional exhaustion, cynicism, and low personal efficacy. The Brief Resilience Scale [10], Cognitive and Affective Mindfulness Scale–Revised [11], and the Self Compassion Scale—Short Form [12] were used to measure qualities that protect against burnout, including resilience, mindfulness, and self-compassion, respectively. Questionnaires were disseminated via email to interested learners on a voluntary basis. All survey responses were anonymous, as no identifying demographic information was collected. Surveys were collected between November 2020 and June 2021, corresponding to the second and third waves of the COVID-19 pandemic.

### Participants

All NOSM University medical students from year one to year four (MS1-4), and all NOSM University residents in their first year of postgraduate training (PGY1) were invited to participate in the associated research study. At the time of survey collection, there were 128 exclusively online learners (MS1 and MS2) and 184 in-person learners (MS3, MS4 and PGY1) who received an invitation to participate in the research study. Demographic information regarding each of these classes were obtained from the NOSM University Undergraduate Medical Education and Postgraduate Education units, respectively, since this data was not obtained in the anonymous surveys. This data is summarized in Table 1.

**Table 1:** Demographics of online and in-person learners.

Learner category	Number of learners	Average age	Male (%)	Female (%)	Other gender (%)	Survey responses (%) ^a^
Online						32%
MS1	64	26	26.6	68.8	4.7	
MS2	64	26	31.2	64.1	4.7	
In-person						18%
MS3	64	27	34.4	65.6	0.0	
MS4	64	28	40.6	59.4	0.0	
PGY1	56	30	42.9	53.6	3.6	

^a^ Survey response percentages are aggregates for all online learners and all in-person learners, as opposed to individually for each year.

### Statistics

Data from questionnaires were converted to numerical scores. Two groups were created for comparative purposes. The first group contained learners who completed their medical education entirely online with no access to in-person learning or clinical exposure during the pandemic. Those who completed exclusively online medical education included first- and second-year medical students. The second group contained learners who continued their medical education in-person, specifically in teaching hospitals and clinics, during the pandemic. This group contained third- and fourth-year medical students, and first-year residents, who were permitted to continue in-person learning. Comparative analyses between those who learned exclusively online and those who continued in-person learning during the pandemic were conducted using T-tests, and significance was set at a value of p < .05. This allowed for identification of differences in burnout (specifically emotional exhaustion, cynicism, and personal efficacy), and protective factors such as resilience, mindfulness, and self-compassion among the two groups who experienced different learning environments during the pandemic. All statistics were carried out using GraphPad Prism 9 [13].

## Results

### Survey responses

Seventy-four respondents completed the questionnaires. Of these, 41 were learners who participated in exclusively online learning during the pandemic, and 33 were learners who were permitted to continue in-person learning at clinics and teaching hospitals throughout the COVID-19 pandemic. These responses were representative of 32% of exclusively online learners and 18% of in-person learners at NOSM University who were sent the survey during that time. These two groups of learners were compared.

### Symptoms of burnout during COVID-19 pandemic

Differences in the frequency of burnout symptoms experienced by learners, depending on their learning environment (i.e., online or in a clinical setting), was elucidated using the data collected from the questionnaires. On average, learners who were exclusively online demonstrated burnout symptoms significantly more often than learners who continued in-person learning in a clinical setting during the COVID-19 pandemic. Specifically, learners who were confined to online learning reported emotional exhaustion and cynicism at a significantly higher frequency than learners who were able to continue learning in a clinical setting throughout the COVID-19 pandemic (Table 2). Learners who participated in exclusively online learning reported symptoms of exhaustion at least once per week, while learners who were permitted to continue in-person learning experienced symptoms of exhaustion less than a few times per month. Those who were learning online also experienced more feelings of cynicism at a frequency of greater than once per month, while those who continued learning in-person in a clinical environment experienced these feelings less than once per month. However, both cohorts reported feelings of personal efficacy at a similarly high frequency of more than once per week.

**Table 2:** Comparison of survey outcomes between online and in-person learners.

Metric	Online learners			In-person learners			*p*
	Mean	Min	Max	Mean	Min	Max	
Self-compassion ^a^	2.927	1.64	4.36	2.967	1.83	4.83	0.8128
Mindfulness ^b^	31.41	18	46	32.30	21	47	0.5336
Resilience ^c^	3.410	2.0	5.0	3.495	2.2	5.0	0.6221
Exhaustion ^d^	4.302	0.8	6.0	2.869	0.8	5.9	<0.0001****
Cynicism ^d^	2.659	0.0	6.0	1.744	0.0	4.2	0.0096**
Personal efficacy ^d^	4.480	2.5	6.0	4.659	2.5	5.8	0.3834

*p<0.05.

**p<0.01.

***p<0.001.

****p<0.0001.

^a^ Self-compassion scale: 1.0–2.5 = low, 2.5–3.5 = normal, 3.5–5.0 = high

^b^ Mindfulness scale: score ranges from 12–48, with higher scores indicating higher levels of mindfulness.

^c^ Brief resilience scale: 1.00–2.99 = low, 3.00–4.30 = normal, 4.31–5.00 = high

^d^ Maslach burnout inventory: 0 = never, 1 = a few times a year or less, 2 = once a month, 3 = a few times a month, 4 = once a week, 5 = a few times a week, 6 = every day

### Protective qualities equal among medical learners in online and in-person learning environments

Despite exclusively online learners experiencing symptoms of burnout at significantly higher frequencies, this group displayed equal levels of resilience, mindfulness, and self-compassion compared to those who continued in-person learning in a clinical environment (Table 2).

## Discussion

This study aimed to demonstrate that medical learners who transitioned to exclusively online learning during the COVID-19 pandemic exhibited higher levels of burnout compared to those who continued in-person, clinical learning and participated in the COVID-19 response. These results confirm the hypothesis that NOSM University learners who were exclusively online learning during the pandemic experienced symptoms of burnout significantly more often than those learners who continued in-person learning during the COVID-19 pandemic, thereby following similar trends observed globally. This trend was observed despite equal levels of protective qualities including resilience, mindfulness, and self-compassion reported between the two groups, suggesting that learners who were exclusively online were equally as equipped as those who continued in-person clinical learning to tolerate circumstances that may lead to burnout. It is postulated that this phenomenon is the result of changes in the learning environment during the pandemic. Although our study is limited to observation of this phenomenon, we cannot provide evidence of a direct causation between online learning and burnout given the limitations of our study. As such, we attempted to provide explanations as to why we observed differing levels of burnout among our learners at NOSM University using existing evidence from the literature.

The results from this study demonstrate that NOSM University learners surveyed have similar levels of protective factors, including resilience, mindfulness, and self-compassion. The available literature suggests an inverse relationship between these factors and burnout [14, 15]. Research suggests that an increase in these factors support an individual’s ability to cope with stressful events and promote emotional balance, thereby protecting against burnout [14, 15]. Given the equivalent levels of protective factors among the two cohorts, our data suggests that a lack of protective factors, including resilience, is not associated with the observed increase in burnout among NOSM University learners. Therefore, there must be another explanation for the high levels of burnout among online learners observed in this study.

Social engagement is a well-established protective factor that is impacted by virtual learning and may contribute to the difference in levels of burnout between in-person and online learners. While social engagement was not studied directly in this case, the support from the literature and relevance to the lives of learners in the context of COVID-19 pandemic warrant some consideration. Social interaction has been established as an important protective factor against physician burnout [16]. As medical education moved online and public health guidelines restricted physical gathering, exclusively online learners became increasingly isolated and lacking social contact with classmates, mentors, and peers, potentially leaving them more susceptible to symptoms of burnout. It is possible that in-person learners benefited from continued social interaction with colleagues and patients, and were potentially protected from symptoms of burnout by this regular social engagement. Further studies on social connection are necessary to delineate the impact on learners and ascertain the degree to which they influence burnout in this setting.

Meaning of work may also have contributed to the differences in burnout observed in our cohort. Studies have demonstrated that physicians who have a sense of meaning in their lives have lower levels of burnout [17]. In the context of the pandemic, the learners who continued to work in clinics and hospitals and participated in the pandemic response likely had a greater sense of meaning in life compared to students who were learning from home and not involved in the pandemic response.

An additional factor that may have contributed to reduced symptoms of burnout among in-person learners may be the reduced hours for many primary care providers and specialists who were not part of the frontline response throughout the COVID-19 pandemic. For learners in these environments, their workload and working hours were often significantly reduced, resulting in a reduction in burnout [18, 19]. This change in the typical structure of the clinical education setting and workload may have contributed to the discrepancy between in-person learners and online learners.

### Study limitations

The results reported were incidental findings, a limitation of the analysis. As a result of the incidental nature, there was no follow-up exploration regarding the participants’ experiences with burnout such as why they were experiencing symptoms of burnout. It is also important to acknowledge that there are differences beyond the pandemic between pre-clerkship and clerkship learners, and residents, including workload and assessment requirements. Additionally, participants were recruited on a voluntary basis from a small medical school in Northern Ontario, a limited sampled that may impact the generalizability of the results. Finally, it should also be noted that there is a lack of baseline scores for pre-pandemic burnout. Future studies should explore possible explanations including social connectedness as protective factors that may contribute to reduced symptoms of burnout, or examine other possible explanations including differences in workload and working hours in the different learning settings.

The results of this study are significant as the COVID-19 pandemic in Canada persists. Furthermore, medical schools continue to discuss the role of online learning post-pandemic. Aligning with these discussions, medical schools across the country will continue to have to make decisions balancing learning objectives, public health guidelines, and student well-being. The promotion of burnout by online learning pedagogy should be heavily considered in the decision-making process to support the mental health and well-being of medical learners across Canada.

## Conclusion

With no clear end to the global COVID-19 pandemic, it is crucial to understand the impact of the online learning environment on the well-being of medical learners. The results discussed in this paper suggest that an increased reliance on online learning during the COVID-19 pandemic may significantly increase the rate of burnout among medical learners, compared to learners who were able to continue learning in a clinical setting. While the results suggest an association between burnout and online learning, the conclusions of this work are limited due to the incidental nature of the finding and the fact that our initial study on mindful self-compassion was not designed to answer this question. Future research should further explore with more detail the phenomena observed in this study and will likely provide additional insight into whether online learning is directly correlated with increased levels of burnout. As medical education continues to incorporate online learning as the COVID-19 pandemic persists and potentially beyond, we must consider the implications of these policy decisions on the well-being of learners.

## Supporting information

S1 Data (XLSX)
